# Reviewed article: Gonçalves SG, Mesas AE, Bravo DS, Birolim MM, Guidoni CM, Nunes EFPA, Nierotka P, Rodrigues R. Effect of work organization and demands, violence, health problems and job satisfaction in relation to work stress and social support in prison officers: a cross-sectional study in penitentiaries in the state of São Paulo, 2019. Epidemiol Serv Saude. 2025:34;e20240714.

**DOI:** 10.1590/S2237-96222025v34e20240714.a

**Published:** 2025-08-11

**Authors:** Virgínia Carvalho

**Table:** 

Rating	Excellent	Good	Average	Below average	Poor
Interest		✓			
Quality				✓	
Originality				✓	
Overall					✓

**Table:** 

Questionnaire	Yes	No	Not applicable
Does the manuscript contain new and significant information to justify publication?		✓	
Does the Abstract (Summary) clearly and accurately describe the content of the article?		✓	
Is the problem significant and concisely stated?		✓	
Are the methods described comprehensively?		✓	
Are the interpretations and conclusions justified by the results?		✓	
Is adequate reference made to other work in the field?		✓	

## Manuscript Structure

Length of article is: Too short

Number of tables is: Adequate

Number of figures is: Adequate

Please state any conflict(s) of interest that you have in relation to the review of this paper (state “none” if this is not applicable): None

## Comments

Com meus cumprimentos aos/às autores/as pela escolha do tema, trago alguns apontamentos que indicam aspectos a serem aprimorados no trabalho, conforme seguem:

1 - Título

O título não esclarece muito bem do que trata o trabalho. A expressão “variáveis relacionadas ao trabalho” é de tamanha abrangência. Inclui um sem número de possibilidades e por essa razão não esclarece devidamente a que variáveis o trabalho se refere. Os autores precisam ser mais específicos para melhor demarcar do que trata o trabalho.

2 - Resumo

O resumo igualmente carece de clareza, a começar pelo objetivo apresentado, que incorre no mesmo problema do título ao tratar de “variáveis ocupacionais”, que é uma expressão equivalente a “variáveis relacionadas ao trabalho” e deixa o leitor sem uma posição mais clara sobre quais variáveis os autores se referem. O método não se encontra suficientemente bem descrito e os resultados apresentam erros de construção textual, com repetidas falhas de concordância nominal. Ainda, a conclusão indicada não esclarece a contribuição do estudo.

3 – Introdução

Nas linhas 41 e 42 da página 2, os autores apontam que “uma das vertentes mais aceitas entende que o estresse ocupacional resulta do sofrimento ou tensão psicológica que surge dos estressores individuais e organizacionais no ambiente de trabalho”. Importante esclarecer a que vertente, mais exatamente, se referem.

Nas linhas 49 a 53 da página 2, afirmam que “Compreendendo a importância do tema, o estresse ocupacional é comumente empregado como variável independente, na qual indivíduos com maiores demandas de trabalho e menor controle sobre este processo, i.e., em trabalho de alta exigência (11,12), estão mais sujeitos ao estresse ocupacional (13)”. Necessário indicar com base em quais evidências os autores afirmam que os estresse ocupacional é comumente empregado como variável independente, haja vista que há um grande número de estudos que aborda o estresse ocupacional numa perspectiva de interação (e em algumas delas de transação) em que são avaliadas as relações entre estímulos e respostas (ou seja, variáveis independentes/estressores e variáveis dependentes/reações).

Nas linhas 9 a 11 da página 3, sustentam que “a maioria dos estudos realizados com policiais penais são realizados em países com condições do sistema carcerário muito distintas das brasileiras como nos Estados Unidos e Europa”. Do que tratam esses estudos? Sobre estresse ocupacional? Quais são os enfoques que adotam e os principais achados? Tais questões devem ser trazidas na introdução de modo a contextualizar o estudo perante o cenário internacional. Outrossim, deve ser contextualizado perante o cenário nacional, indicando do que trata a produção brasileira sobre o tema e quais as potenciais contribuições do referido estudo perante o cenário apresentado.

4 – Método

O estudo deve indicar, no método, o número de participantes e se a amostra utilizada foi probabilística (se sim, de que tipo) ou não probabilística (e de que natureza). Deve apontar ainda as propriedades psicométricas da medida adotada para a mensuração de estressores.

5 – Resultados

Informações que se encontram no primeiro parágrafo dos resultados deveriam constar no método. O estudo confunde os conceitos de população e amostra.

Há certa inconsistência entre a proposta do estudo e algumas análises empreendidas, haja vista que avaliou a relação entre variáveis sociodemográficas e percepção de estressores, sendo que estado civil e idade não são “variáveis ocupacionais”.

6 - Discussão

O fato de não haverem explorado devidamente a literatura nacional e internacional sobre o tema (estresse laboral junto a policiais penais) prejudicou a discussão dos resultados que trata o tema sem considerar tudo o que já foi produzido sobre o assunto e sem cotejar os resultados à luz de referida literatura.

Nas linhas 22 e 23 da página 8 a pesquisa se classifica como censitária, o que entra em contradição com o que foi apontado no método, uma vez que lá é indicado que houve agentes que não foram entrevistados.

Por fim, é importante que sejam elencadas as contribuições do estudo perante a literatura da área, bem como maiores esclarecimentos sobre as limitações do estudo.

